# Transient Gel Electrophoresis of a Spherical Colloidal Particle

**DOI:** 10.3390/gels9050356

**Published:** 2023-04-23

**Authors:** Hiroyuki Ohshima

**Affiliations:** Faculty of Pharmaceutical Sciences, Tokyo University of Science, 2641 Yamazaki, Noda, Chiba 278-8510, Japan; ohshima@rs.noda.tus.ac.jp

**Keywords:** transient gel electrophoresis, transient electrophoresis, gel electrophoresis

## Abstract

The general theory is developed for the time-dependent transient electrophoresis of a weakly charged spherical colloidal particle with an electrical double layer of arbitrary thickness in an uncharged or charged polymer gel medium. The Laplace transform of the transient electrophoretic mobility of the particle with respect to time is derived by considering the long-range hydrodynamic interaction between the particle and the polymer gel medium on the basis of the Brinkman–Debye–Bueche model. According to the obtained Laplace transform of the particle’s transient electrophoretic mobility, the transient gel electrophoretic mobility approaches the steady gel electrophoretic mobility as time approaches infinity. The present theory of the transient gel electrophoresis also covers the transient free-solution electrophoresis as its limiting case. It is shown that the relaxation time for the transient gel electrophoretic mobility to reach its steady value is shorter than that of the transient free-solution electrophoretic mobility and becomes shorter as the Brinkman screening length decreases. Some limiting or approximate expressions are derived for the Laplace transform of the transient gel electrophoretic mobility.

## 1. Introduction

When an external electric field is suddenly applied to a suspension of colloidal particles, the particle starts to move with a time-dependent transient electrophoretic mobility, which reaches steady electrophoretic mobility as time goes to infinity. While there are many theoretical studies on transient free-solution electrokinetics [1,2,3,4,5,6,7,8,9,10,11,12,13,14,15,16,17], including transient electrophoresis of spherical hard particles [1,4,6,7,9,13,14,17], cylindrical hard particles [2,11,16] and soft particles (i.e., polyelectrolyte-coated particles) [12,15], and on steady gel electrophoresis [18,19,20,21,22,23,24,25,26,27,28,29,30,31,32,33,34,35,36,37,38,39] including gel electrophoresis of spherical hard particles [18,19,20,21,22,23,24,25,28,30,31,32,34,35], soft particles [26,27,29,33,36,37,38] and liquid droplets [39], there are only a few theoretical studies on the transient gel electrophoresis by Saad and Faltas [40], Saad [41,42], and Sherief, et al., [43]. In the present paper, we further develop the theory of Saad and Faltus [40] and present the general theory of transient gel electrophoresis of a weakly charged, spherical solid colloidal particle with an electrical double layer of arbitrary thickness. There are two types of interactions between the particle and the gel medium: (i) the short-range steric interaction due to the particle-gel friction, and (ii) the long-range hydrodynamic interaction. For dilute gels, where the particle size is much smaller than the gel pore size, the long-range hydrodynamic interaction becomes dominant. In this paper, we treat a dilute gel medium and consider the long-range hydrodynamic interaction between the particle and the polymer gel medium on the basis of the Brinkman–Debye–Bueche model [44,45] and derive an expression for the Laplace transform of the transient electrophoretic mobility of a spherical solid colloidal particle. From the obtained Laplace transform of the transient gel electrophoretic mobility, the transient gel electrophoretic mobility can be derived numerically by using the inverse Laplace transformation.

## 2. Theory

### 2.1. Fundamental Electrokinetic Equations

Consider a charged spherical colloidal particle of radius *a* and relative permittivity *ε*_p_, carrying zeta potential *ζ* in a charged polymer gel medium containing an electrolyte solution of viscosity *η* and relative permittivity *ε*_r_. The Brinkman–Debye–Bueche continuum medium [44,45] is employed, in which polymer segments are considered to be resistance centers, exerting frictional forces on the liquid flowing through the gel medium. The gel medium is regarded as a uniform continuum medium, which contains fixed charges of density *ρ*_fix_, free mobile electrolyte ions of density *ρ*_el_(***r***) at position ***r***, including added electrolyte ions and gel counterions. Let the electrolyte be composed of *N* ionic species of valence *z_i_*, bulk concentration (number density) $n_{i}^{\infty }$ and drag coefficient *Λ_i_* (*i* = 1, 2, …, *N*), and the gel counterions be of *N* + 1-th ionic species of valence *z_N+1_*, bulk concentration (number density) $n_{N+1}^{\infty }$ and drag coefficient *Λ_N_*_+1_. The electroneutrality condition of the system is given by

(1) $$\sum_{i=1}^{N+1}z_{i}{en}_{i}^{\infty }+\rho_{fix}=0$$

where *e* is the elementary electric charge.

**Figure 1:**
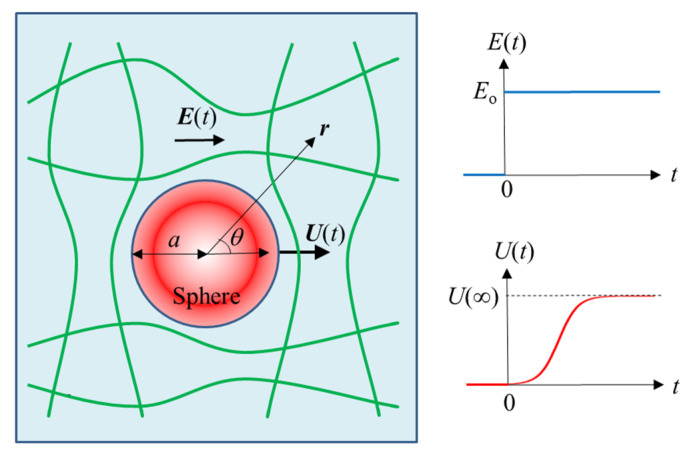
Spherical colloidal particle of radius *a* and zeta potential *ζ* moving with transient electrophoretic velocity ***U***(*t*) in a polymer gel medium under an applied step electric field ***E***(*t*). *U*(∞) is the magnitude of the static electrophoretic velocity at *t* = ∞.

We suppose that at time *t* = 0, a step electric field ***E***(*t*) is suddenly applied to the particle, viz.,

(2) $$E(t)=\left\{\begin{array}{cc} 0, & t=0\\ E_{o}, & t>0 \end{array}\right.$$

where ***E***_o_ is a constant. The particle then starts to migrate with an electrophoretic velocity ***U***(*t*) (*U*(*t*)cos*θ*, −*U*(*t*)sin*θ*, 0) in the direction parallel to ***E***_o_, *U*(*t*) being the magnitude of ***U***(*t*) (Figure 1). 

Our model uses a frame of reference fixed at the center of the particle. The origin of the coordinate system (*r*, *θ*, *ϕ*) is held fixed at the particle center, and the polar axis (*θ* = 0) is set parallel to ***E*** (*t*). The transient electrophoretic mobility *μ*(*t*) of the particle is defined by ***U***(*t*) = *μ*(*t*)***E***(*t*) = *μ*(*t*)***E***_o_. Our model treats the case in which the following conditions are fulfilled: (i) the liquid in the gel medium can be considered to be incompressible; (ii) the applied electric field ***E***(*t*) is so weak that the particle velocity ***U***(*t*) is proportional to ***E***(*t*), and terms involving the square of the liquid velocity in the Navier–Stokes equation can be neglected in our model; (iii) the slipping plane, at which the liquid velocity ***u***(***r***, *t*) relative to the particle is zero, is located on the particle surface (at *r* = *a*); (iv) electrolyte ions cannot penetrate the particle surface [46]; and (v) in equilibrium (in the absence of ***E***(*t*)), the ion distribution is assumed to be given by the Boltzmann distribution and the electric potential follows the Poisson–Boltzmann equation. 

Under these conditions (i)–(v), the fundamental electrokinetic equations for the liquid flow velocity ***u***(***r***, *t*) (*u_r_*(***r***, *t*), *u_θ_*(***r***, *t*), 0) at position ***r***(*r*, *θ*, *ϕ*) and time *t* and the velocity ***v****_i_*(***r***, *t*) of *i* th ionic species are given by.

(3) $$\rho_{o}\frac{\partial }{\partial t}\left\{u\left(r,t\right)+U(t)\right\}+\eta \nabla \times \nabla \times u\left(r,t\right)+\nabla p\left(r,t\right)+\rho_{el}\left(r,t\right)\nabla \psi \left(r,t\right)+\gamma \left(u\left(r,t\right)+U(t)\right)=0$$

(4) $$\nabla \cdot u\left(r,t\right)=0$$

(5) $$v_{i}\left(r,t\right)=u\left(r,t\right)-\frac{1}{\Lambda_{i}}\nabla \mu_{i}\left(r,t\right)$$

(6) $$\frac{\partial n_{i}\left(r,t\right)}{\partial t}+\nabla \cdot \left\{n_{i}\left(r,t\right)v_{i}\left(r,t\right)\right\}=0$$

with

(7) $$\rho_{el}\left(r,t\right)=\sum_{i=1}^{N+1}z_{i}n_{i}\left(r,t\right)$$

(8) $$\mu_{i}\left(r,t\right)=\mu_{i}^{o}+z_{i}e\psi \left(r,t\right)+kTln\left[n_{i}\left(r,t\right)\right]$$

(9) $$∆\psi \left(r,t\right)=-\frac{\rho_{el}\left(r,t\right)}{\varepsilon_{r}\varepsilon_{o}}$$

where *k* is the Boltzmann constant, *T* is the absolute temperature, *ε*_o_ is the permittivity of a vacuum, *p*(***r***, *t*) is the pressure, *ρ*_el_(***r***, *t*) is the charge density and *ψ*(***r***, *t*) is the electric potential. Equation (3) is the Navier–Stokes equation, and Equation (4) is the equation of continuity for an incompressible flow (condition (i)). The term involving ***U*** (*t*) in Equation (3) arises from the fact that the particle has been chosen as the frame of reference for the coordinate system. Equation (5) means that the flow ***v**_i_*(***r***, *t*) of the *i* th ionic species is caused by ***u***(***r***, *t*), and the gradient of the electrochemical potential *μ_i_*(***r***, *t*), given by Equation (8), in which $\mu_{i}^{o}$ is a constant term. Equation (6) is the continuity equation for the *i* th ionic species. Equation (9) is the Poisson equation. Note that in the absence of the particle, there exists a time-dependent transient electroosmotic flow, which is parallel to ***E***(*t*). The transient electroosmotic flow velocity ***u***_EOF_(*t*) = (*u*_EOF_(*t*)cos*θ*, −*u*_EOF_(*t*)sin*θ*, 0) obeys

(10) $$\rho_{o}\frac{\partial }{\partial t}u_{EOF}\left(t\right)+\rho_{fix}E\left(t\right)+\gamma u_{EOF}\left(t\right)=0$$

where *u*_EOF_(*t*) is the magnitude of ***u***_EOF_(*t*). 

The following initial condition and boundary conditions at the particle surface (at *r* = *a*) and far from the particle (*r* → ∞) must be satisfied:

(11) $$u\left(r,t\right)=0\;\mathrm{at}\;t=0$$

(12) $$u\left(r,t\right)=0\;at\;r=a$$

(13) $$u\left(r,t\right)\to -U\left(t\right)+u_{EOF}(t)\;at\;as\;r\to \infty$$

(14) $$v_{i}\left(r,t\right)\cdot \hat{n}=0\;at\;r=a$$

(15) $$\psi \left(r,t\right)\to -E\left(t\right)\cdot r\;as\;r\to \infty$$

where $\hat{n}$ is the unit normal outward from the particle surface. Equation (12) is the no-slip boundary condition at the particle surface (condition (iii)). Equation (14) is derived from condition (iv). Equation (15) implies that *ψ*(***r****, t*) tends to the potential of the applied electric field ***E***(*t*) as *r* →∞. 

In addition, the particle velocity ***U***(*t*) obeys the following equation of motion of the particle:

(16) $$\frac{{4\pi a}^{3}}{3}\rho_{p}\frac{dU(t)}{dt}=F_{H}(t)+F_{E}(t)$$

where ***F***_H_(*t*) and ***F***_E_(*t*) are, respectively, the hydrodynamic and electric forces acting on the particle and are defined by

(17) $$F_{H}(t)=\int_{0}^{\pi }{\left[\left(-p+2\eta \frac{\partial u_{r}}{\partial r}\right)cos\theta -\eta \left(\frac{1}{r}\frac{\partial u_{r}}{\partial \theta }+\frac{\partial u_{\theta }}{\partial r}-\frac{u_{\theta }}{r}\right)sin\theta \right]}_{r=a}2\pi a^{2}sin\theta d\theta \frac{E(t)}{E_{o}}$$

(18) $$F_{E}(t)=\varepsilon_{r}\varepsilon_{o}\int_{0}^{\pi }{\left[\left\{\frac{\partial \psi }{\partial r}\left(\frac{\partial \psi }{\partial r}cos\theta -\frac{1}{r}\frac{\partial \psi }{\partial \theta }sin\theta \right)\right\}-\frac{1}{2}\left\{{\left(\frac{\partial \psi }{\partial r}\right)}^{2}+{\left(\frac{1}{r}\frac{\partial \psi }{\partial \theta }\right)}^{2}\right\}cos\theta \right]}_{r=a}2\pi a^{2}sin\theta d\theta \frac{E(t)}{E_{o}}$$

Equation (16) serves as a boundary condition for ***u***(***r***, *t*).

### 2.2. Weak Electric Field Approximation

For a weak electric field ***E***(*t*), the deviations of *n_j_*(***r****, t*), *ψ*(***r****, t*) and *μ_j_*(***r****, t*) from their equilibrium values due to ***E***(*t*) are all small so that we may write

(19) $$n_{i}\left(r,t\right)=n_{i}^{\left(0\right)}\left(r\right)+\delta n_{i}\left(r,t\right)$$

(20) $$\psi \left(r,t\right)=\psi^{\left(0\right)}\left(r\right)+\delta \psi \left(r,t\right)$$

(21) $$\mu_{i}\left(r,t\right)=\mu_{i}^{\left(0\right)}+\delta \mu_{i}\left(r,t\right)$$

where the quantities with superscript (0) refer to the equilibrium values and $\mu_{i}^{\left(0\right)}$ is a constant independent of *r*. The equilibrium concentration $n_{i}^{\left(0\right)}\left(r\right)$ is assumed to be given by the Boltzmann distribution, and the equilibrium electric potential obeys the Poisson–Boltzmann equation (condition (v)), viz.,

(22) $$n_{i}^{\left(0\right)}\left(r\right)=n_{i}^{\infty }exp\left(-\frac{z_{i}e\psi^{\left(0\right)}\left(r\right)}{kT}\right)$$

(23) $$∆\psi^{\left(0\right)}\left(r\right)=-\frac{\rho_{el}^{\left(0\right)}\left(r\right)+\rho_{fix}}{\varepsilon_{r}\varepsilon_{o}}$$

(24) $$\rho_{el}^{\left(0\right)}\left(r\right)=\sum_{i=1}^{N+1}z_{i}en_{i}^{\left(0\right)}\left(r\right)=\sum_{i=1}^{N+1}z_{i}en_{i}^{\infty }exp\left(-\frac{z_{i}e\psi^{\left(0\right)}\left(r\right)}{kT}\right)$$

The boundary conditions for *ψ*^(0)^(*r*) are given by

(25) $$\psi^{\left(0\right)}\left(a\right)=\zeta$$

(26) $$\psi^{\left(0\right)}\left(r\right)\to 0\;as\;r\to \infty$$

By substituting Equations (19)–(21) into Equation (3) and neglecting the products of the small quantities, we finally obtain

(27) $$\rho_{o}\frac{\partial }{\partial t}\nabla \times u\left(r,t\right)+\eta \nabla \times \nabla \times \nabla \times u\left(r,t\right)+\gamma \nabla \times u\left(r,t\right)+\sum_{i=1}^{N+1}\nabla n_{i}^{\left(0\right)}\left(r\right)\times \nabla \delta \mu_{i}\left(r\right)=0$$

and form Equation (6)

(28) $$\frac{\partial }{\partial t}\delta n_{i}\left(r,t\right)+\nabla \cdot \left\{n_{i}^{\left(0\right)}\left(r\right)u\left(r,t\right)-\frac{1}{\Lambda_{i}}n_{i}^{\left(0\right)}\left(r\right)\nabla \delta \mu_{i}\left(r,t\right)\right\}=0$$

Further, from symmetry, we may write

(29) $$u\left(r,t\right)=\left(-\frac{2}{r}h\left(r,t\right)E(t)cos\theta ,\frac{1}{r}\frac{\partial }{\partial r}(rh\left(r,t\right))E(t)sin\theta ,0\right)$$

(30) $$\delta \mu_{i}\left(r,t\right)=-z_{i}e\phi_{i}\left(r,t\right)E(t)cos\theta$$

(31) $$\delta \psi \left(r,t\right)=-Y\left(r,t\right)E(t)cos\theta$$

where *E*(*t*) is the magnitude of ***E***(*t*), and *h*(*r, t*), *ϕ_i_*(*r*, *t*) and *Y*(*r*, *t*) are functions of *r* and *t*. By substituting Equations (29)–(31) into Equations (27) and (28), we obtain the following equations for *h*(*r*) and *ϕ_i_*(r), and *Y*(*r*):

(32) $$L\left[Lh(r,t)-\lambda^{2}h(r,t)-\frac{1}{\nu }\frac{\partial h(r,t)}{\partial t}\right]=G\left(r,t\right)$$

(33) $$L\phi_{i}(r,t)-\frac{\Lambda_{i}}{kT}\frac{\partial }{\partial t}\left\{\phi_{i}(r,t)-Y(r,t)\right\}=\frac{dy(r)}{dr}\left\{z_{i}\frac{\partial \phi_{i}(r,t)}{\partial r}-\frac{2\lambda_{i}}{e}\frac{h(r,t)}{r}\right\}$$

(34) $$LY(r,t)=\frac{e^{2}}{\varepsilon_{r}\varepsilon_{o}kT}\sum_{i=1}^{N+1}z_{i}^{2}n_{i}^{\infty }e^{-z_{i}y\left(r\right)}\left\{Y\left(r,t\right)-\phi_{i}(r,t)\right\}$$

with

(35) $$y(r)=\frac{e\psi^{\left(0\right)}(r)}{kT}$$

(36) $$\lambda ={\left(\frac{\gamma }{\eta }\right)}^{1/2}$$

where the scaled equilibrium electric potential *y*(*r*) is introduced, *λ* is the reciprocal of the Brinkman screening length 1/*λ*,

(37) $$L=\frac{\partial }{\partial r}\frac{1}{r^{2}}\frac{\partial }{\partial r}r^{2}=\frac{\partial^{2}}{\partial r^{2}}+\frac{2}{r}\frac{\partial }{\partial r}-\frac{2}{r^{2}}$$

is a differential operator, and *G*(*r*, *t*) is defined by

(38) $$G\left(r,t\right)=-\frac{e}{\eta r}\frac{dy}{dr}\sum_{i=1}^{N}z_{i}^{2}n_{i}^{\infty }e^{-z_{i}y}\phi_{i}\left(r,t\right)$$

and

(39) $$\nu =\frac{\eta }{\rho_{o}}$$

is the kinematic viscosity. 

### 2.3. General Expression for the Laplace Transform of the Transient Gel Electrophoretic Mobility 

The transient electrophoretic mobility *μ*(*t*) can be obtained from Equation (13), viz.,

(40) $$\mu \left(t\right)=\frac{U(t)}{E(t)}=\frac{U(t)}{E_{o}}=2\underset{r\to \infty }{\lim}\frac{h(r,t)}{r}+u_{}(t)$$

Here *h*(*r*, *t*) is the solution to Equation (32), which can be most easily solved by using the Laplace transformation with respect to time *t*. The Laplace transforms $\hat{h}\left(r,s\right)$, ${\hat{u}}_{EOF}\left(s\right)$, $\hat{G}\left(r,s\right)$ and $\hat{\mu }\left(s\right)$ of *h*(*r*, *t*), *u*_EOF_(*t*), *G*(*r*, *t*) and *μ*(*t*), respectively, are defined by

(41) $$\hat{h}\left(r,s\right)=\int_{0}^{\infty }h\left(r,t\right)e^{-st}dt$$

(42) $${\hat{u}}_{EOF}\left(s\right)=\int_{0}^{\infty }u_{EOF}\left(t\right)e^{-st}dt$$

(43) $$\hat{Y}\left(r,s\right)=\int_{0}^{\infty }Y\left(r,t\right)e^{-st}dt$$

(44) $$\hat{G}\left(r,s\right)=\int_{0}^{\infty }G\left(r,t\right)e^{-st}dt$$

Thus, the Laplace transform of Equation (32) yields

(45) $$L\left[L\hat{h}(r,s)-\lambda^{2}\hat{h}(r,s)-\frac{s}{\nu }\hat{h}(r,s)\right]=\hat{G}\left(r,s\right)$$

which is solved to give

$$\hat{h}(r,s)=-\frac{1}{3\beta^{2}}\int_{\infty }^{r}\left(r-\frac{x^{3}}{r^{2}}\right)\hat{G}\left(x,s\right)dx$$

$$-\frac{1}{\beta^{3}}\int_{\infty }^{r}\left\{\left(\frac{x}{\beta r^{2}}-\frac{1}{\beta r}\right)\cosh\left[\beta \left(r-x\right)\right]-\left(\frac{x}{r}-\frac{1}{\beta^{2}r^{2}}\right)\sinh\left[\beta \left(r-x\right)\right]\right\}\hat{G}\left(x,s\right)dx$$

(46) $$-\frac{C_{1}r}{\beta^{2}}-\frac{C_{2}}{\beta^{2}r^{2}}+C_{3}\left(\frac{\beta }{r}+\frac{1}{r^{2}}\right)e^{-\beta (r-a)}$$

with

(47) $$\beta =\sqrt{\lambda^{2}+\frac{s}{\nu }}$$

where *C*_1_–*C*_3_ are integration constants to be determined.

From the Laplace transform of Equation (10), we obtain

(48) $${\hat{u}}_{EOF}\left(s\right)=-\frac{\rho_{fix}}{\eta \beta^{2}s}E_{o}$$

Equation (40) for the Laplace transform of the transient gel electrophoretic mobility *μ*(*t*) thus becomes

(49) $$\hat{\mu }(s)=2\underset{r\to \infty }{\lim}\frac{\hat{h}(r,s)}{r}-\frac{\rho_{fix}}{\eta \beta^{2}s}$$

By determining the integration constants *C*_1_–*C*_3_ in Equation (46) to satisfy the boundary conditions (Equations (11)–(16)) and using Equation (40), we finally obtain the following expression for the Laplace transform $\hat{\mu }\left(s\right)$ of the transient gel electrophoretic mobility *μ*(*t*) of a sphere:

$$\hat{\mu }(s)=\frac{2}{3\beta^{2}\Omega }\int_{a}^{\infty }\left\{-\left(1+\beta a+\frac{\beta^{2}a^{2}}{3}\right)+\left(1+\beta r\right)e^{-\beta \left(r-a\right)}+\frac{\beta^{2}r^{3}}{3a}\right\}\hat{G}\left(r,s\right)dr$$

(50) $$-\frac{\rho_{fix}}{\eta \beta^{2}\Omega s}\left\{1+\beta a+\frac{\beta^{2}a^{2}}{3}-\frac{{2\beta }^{2}as}{9}\hat{Y}\left(a,s\right)\right\}$$

with

(51) $$\Omega =1+\beta a+\frac{\beta^{2}a^{2}}{9}+\frac{2\rho_{p}}{9\rho_{o}}\frac{a^{2}}{\nu }s$$

## 3. Results and Discussion

Equation (50) is the required general expression for $\hat{\mu }\left(s\right)$, which is applicable for arbitrary values of the particle zeta potential *ζ* and *κa*. The transient electrophoretic mobility *μ*(*t*) can be obtained numerically from Equation (50) by the inverse transform method. 

Consider the following two limiting cases. In the limit of *t* → ∞, *μ*(*t*) tends to the steady gel electrophoretic mobility *μ*(∞) = *μ*_s_, which can be obtained from $\hat{\mu }\left(s\right)$ by using the following formula:

(52) $$\mu_{s}=\mu \left(\infty \right)=\underset{s\to 0}{\lim}\left[s\hat{\mu }(s)\right]$$

The result is

(53) $$\mu_{s}=\frac{2}{3\lambda^{2}\Omega_{s}}\int_{a}^{\infty }\left\{-\left(1+\lambda a+\frac{\lambda^{2}a^{2}}{3}\right)+\left(1+\lambda r\right)e^{-\lambda \left(r-a\right)}+\frac{\lambda^{2}r^{3}}{3a}\right\}G(r)dr-\frac{\rho_{fix}}{\eta \lambda^{2}}\left[1-\frac{{2\lambda }^{2}a^{2}}{9\Omega_{s}}\left\{\frac{Y(a)}{a}-1\right\}\right]$$

with

(54) $$\Omega_{s}=1+\lambda a+\frac{\lambda^{2}a^{2}}{9}$$

Equation (53) agrees with the general expression for the steady electrophoretic mobility m(t) of a sphere in a polymer gel medium [35]. Next, in the limit of *ρ*_fix_ = 0 and *λ* = 0, i.e., *β* = $\sqrt{s/\nu }$), Equation (50) reduces to

(55) $$\hat{\mu }(s)=\frac{2\nu }{3s\Omega_{f}}\int_{a}^{\infty }\left\{-\left(1+a\sqrt{\frac{s}{\nu }}+\frac{a^{2}s}{3\nu }\right)+\left(1+\sqrt{\frac{s}{\nu }}r\right)exp\left[-\sqrt{\frac{s}{\nu }}\left(r-a\right)\right]+\frac{r^{3}s}{3a\nu }\right\}\hat{G}\left(r,s\right)dr$$

with

(56) $$\Omega_{f}=1+a\sqrt{\frac{s}{\nu }}+\frac{a^{2}s}{9\nu }+\frac{2\rho_{p}}{9\rho_{o}}\frac{a^{2}s}{\nu }$$

which agrees with the general expression for the Laplace transform $\hat{\mu }(s)$ of the transient electrophoretic mobility *μ*(*t*) of a sphere in a free solution [14]. It is thus found that in the above two limiting cases, Equation (50) reduces to the correct limiting forms.

Now consider the case where the particle *ζ* potential is low, and the relative permittivity of *ε*_p_ of the particle is much smaller than that of the electrolyte solution *ε*_r_ (*ε*_p_ « *ε*_r_) so that *ε*_p_ is practically equal to zero. In this case, Equations (33) and (34) give

(57) $$\phi_{i}\left(r,t\right)=Y(r,t)=r+\frac{a^{3}}{2r^{2}}$$

and Equation (38) becomes

(58) $$G\left(r,t\right)=-\frac{\varepsilon_{r}\varepsilon_{o}\kappa^{2}}{\eta }\left(1+\frac{a^{3}}{2r^{3}}\right)\frac{d\psi^{\left(0\right)}(r)}{dr}$$

with

(59) $$\kappa =\sqrt{\frac{e^{2}}{\varepsilon_{r}\varepsilon_{o}kT}\sum_{i=1}^{N+1}z_{i}^{2}n_{i}^{\infty }}$$

where *κ* is the Debye–Hückel parameter (1/*κ* is the Debye length). The Laplace transform $\hat{G}\left(r,s\right)$ of *G*(*r*, *t*) is thus given by

(60) $$\hat{G}\left(r,s\right)=\frac{G\left(r,t\right)}{s}=-\frac{\varepsilon_{r}\varepsilon_{o}\kappa^{2}}{\eta s}\left(1+\frac{a^{3}}{2r^{3}}\right)\frac{d\psi^{\left(0\right)}(r)}{dr}$$

where the equilibrium electric potential *ψ*^(0)^(*r*) for low *ζ* potential is given by

(61) $$\psi^{\left(0\right)}(r)=\zeta \frac{a}{r}e^{-\kappa (r-a)}$$

which is obtained from the linearized Poisson–Boltzmann equation ∆*ψ*^(0)^(*r*) = *κ*^2^*ψ*^(0)^(*r*) (see Equation (23)). By substituting Equation (58) into Equation (55), we obtain

(62) $$\hat{\mu }\left(s\right)=\frac{2\varepsilon_{r}\varepsilon_{o}\kappa^{2}}{3\beta^{2}\Omega \eta s}\int_{a}^{\infty }\left\{\left(1+\beta a+\frac{\beta^{2}a^{2}}{3}\right)-\left(1+\beta r\right)e^{-\beta \left(r-a\right)}-\frac{\beta^{2}r^{3}}{3a}\right\}\left(1+\frac{a^{3}}{2r^{3}}\right)\frac{d\psi^{\left(0\right)}(r)}{dr}dr-\frac{\rho_{fix}}{\eta \beta^{2}\Omega s}\left(1+\beta a\right)$$

Equation (62) can be rewritten in terms of exponential integrals as

$$\hat{\mu }\left(s\right)=\frac{{2\varepsilon }_{r}\varepsilon_{o}\zeta }{3\eta \Omega s}\left[1+\frac{\kappa \beta a}{\kappa +\beta }+\frac{3\kappa^{2}}{2\beta^{2}}\left(1+\beta a+\frac{\beta^{2}a^{2}}{3}\right)e^{\kappa a}E_{5}\left(\kappa a\right)\right.$$

$$-\frac{3\kappa^{2}}{2\beta^{2}}e^{\left(\kappa +\beta \right)a}\left.\left\{E_{5}\left(\left(\kappa +\beta \right)a\right)+\beta aE_{4}\left(\left(\kappa +\beta \right)a\right)+\frac{\beta^{2}a^{2}}{3}E_{3}\left(\left(\kappa +\beta \right)a\right)\right\}\right]$$

(63) $$-\frac{\rho_{fix}}{\eta \beta^{2}\Omega s}\left(1+\beta a\right)$$

where *E_n_*(*κa*) is the exponential integral of order *n* and is defined by

(64) $$E_{n}\left(\kappa a\right)={\left(\kappa a\right)}^{n-1}\int_{\kappa a}^{\infty }\frac{e^{-t}}{t^{n}}dt$$

Equations (62) and (63) are the generalization of the result of Saad and Faltas [40] and are applicable for low zeta potentials and arbitrary values of *κa*.

Equations (62) and (63) involve integration or exponential integrals, so they are not very convenient for practical use. To avoid this inconvenience, we approximately replace *r* in the factor (1 + *a*^3^/2*r*^3^) by *r* = *a* + *δ*/, viz.,

(65) $$1+\frac{a^{3}}{2r^{3}}\approx 1+\frac{1}{2{\left(1+\frac{\delta }{\kappa a}\right)}^{3}}$$

In the steady gel electrophoresis [35], we have found that the best approximation can be achieved if *δ* is chosen to be *δ* = (2.33*κ* + 1.52*λ*)/(*κ* + *λ*), and the maximum relative error becomes less than 1.6%. We use this choice of *δ* in the transient gel electrophoresis problem. By using this approximation, the integration in Equation (62) can be carried out analytically to give

(66) $$\hat{\mu }\left(s\right)=\frac{{2\varepsilon }_{r}\varepsilon_{o}\zeta }{3\eta \Omega s}\left(1+\frac{\kappa \beta a}{\kappa +\beta }\right)\left[1+\frac{1}{2{\left\{1+\frac{\left(2.33\kappa +1.52\beta \right)}{(\kappa +\beta )\kappa a}\right\}}^{3}}\right]-\frac{\rho_{fix}}{\eta \lambda^{2}\Omega s}\left(1+\beta a\right)$$

We next consider the following two limiting cases.

(i) In the limit of *κa* → ∞ (Smoluchowski limit), Equation (66) becomes

(67) $$\hat{\mu }\left(s\right)=\frac{\varepsilon_{r}\varepsilon_{o}\zeta (1+\beta a)}{\eta \Omega s}-\frac{\rho_{fix}}{\eta \beta^{2}\Omega s}(1+\beta a)$$

(ii) In the limit of *κa* → 0 (Hückel limit), Equation (66) becomes

(68) $$\hat{\mu }\left(s\right)=\frac{{2\varepsilon }_{r}\varepsilon_{o}\zeta }{3\eta \Omega s}-\frac{\rho_{fix}}{\eta \beta^{2}\Omega s}\left(1+\beta a\right)$$

**Figure 2:**
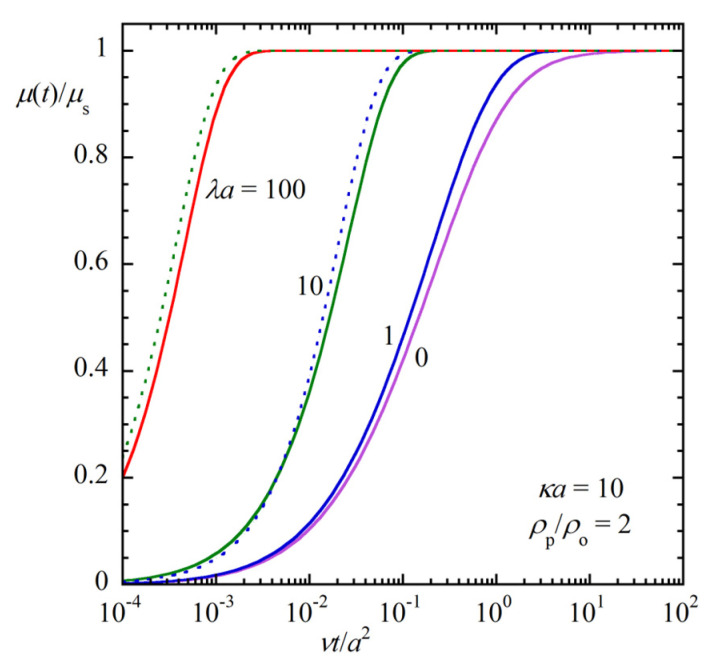
Transient gel electrophoretic mobility *μ*(*t*) of a sphere of radius *a* and mass density *ρ*_p_, carrying zeta potential *ζ* in an uncharged gel medium (*ρ*_fix_ = 0) of the Debye length 1/*κ*, mass density *ρ*_o_ and viscosity *η*. The ratio of *μ*(*t*) at time *t* to its value *μ*(∞) at *t* = ∞, which is equal to the steady gel electrophoretic mobility *μ*_s_ (*μ*(∞) = *μ*_s_), is plotted as a function of the scaled time *νt*/*a*^2^, *ν* being the kinematic viscosity (*ν* = *η*/*ρ*_o_) for the case where *κa* = 10 and *ρ*_p_/*ρ*_o_ = 2). The dotted curves (*λa* = 10, and 100) are the result calculated in Equation (72) for the large *λa* approximate gel electrophoretic mobility.

Figure 2 shows some results of the calculation of the transient gel electrophoretic mobility *μ*(*t*) of a sphere of radius *a*, zeta potential *ζ* and mass density *ρ*_p_ in an uncharged gel medium (*ρ*_fix_=0) of the Debye length 1/*κ*, mass density *ρ*_o_ and viscosity *η*. The ratio of *μ*(*t*) at time *t* to its value *μ*(∞) at *t* = ∞, which is the steady gel electrophoretic mobility *μ*_s_ (*μ*(∞) = *μ*_s_), is plotted as a function of the scaled time *νt*/*a*^2^, *ν* being the kinematic viscosity (*ν* = *η*/*ρ*_o_) for *κa* = 10 and *ρ*_p_/*ρ*_o_ = 2. The transient gel electrophoretic mobility can be obtained numerically from $\hat{\mu }\left(s\right)$ (Equation (63) or Equation (66) with negligible errors) via the inverse Laplace transformation method.

Figure 2 shows that the relaxation time required for *μ*(*t*) to reach its steady value *μ*_s_ becomes shorter as *λa* increases. An approximate expression for the relaxation time *T* for large *λa* can be derived as follows. For large *λa*, *β* in Equation (60) can be approximately replaced with *λ* and Equation (61) reduces to

(69) $$\hat{\mu }\left(s\right)=\frac{2\varepsilon_{r}\varepsilon_{o}\kappa^{2}}{3\lambda^{2}\Omega \eta s}\int_{a}^{\infty }\left\{\left(1+\lambda a+\frac{\lambda^{2}a^{2}}{3}\right)-\left(1+\lambda r\right)e^{-\beta \left(r-a\right)}-\frac{\lambda^{2}r^{3}}{3a}\right\}\left(1+\frac{a^{3}}{2r^{3}}\right)\frac{d\psi^{\left(0\right)}(r)}{dr}dr-\frac{\rho_{fix}}{\eta \lambda^{2}\Omega s}\left(1+\lambda a\right)$$

and we obtain

(70) $$\frac{\hat{\mu }\left(s\right)}{\mu_{s}}=\frac{\Omega_{s}}{s\Omega }=\frac{\Omega_{s}}{s\left(\Omega_{s}+\frac{2\rho_{p}}{9\rho_{o}}\frac{a^{2}}{\nu }s\right)}$$

From Equation (70), the transient gel electrophoretic mobility *μ*(*t*) can be derived, viz.,

(71) $$\frac{\mu (t)}{\mu_{s}}=1-exp\left(-\frac{9\rho_{o}\Omega_{s}}{2\rho_{p}}\frac{\nu t}{a^{2}}\right)$$

which can be rewritten as

(72) $$\frac{\mu (t)}{\mu_{s}}=1-e^{-t/T}$$

with

(73) $$T=\frac{2\rho_{p}a^{2}}{9\rho_{o}\Omega_{s}\nu }$$

Here *T* can be regarded as the relaxation time. The relaxation time *T*_f_ for the transient free-solution electrophoresis is given by

(74) $$T_{f}=\frac{2\rho_{p}a^{2}}{9\rho_{o}\nu }$$

so that

(75) $$\frac{T}{T_{f}}=\frac{1}{\Omega_{s}}=\frac{1}{1+\lambda a+\lambda^{2}a^{2}/9}$$

which shows that the relaxation time *T* for the transient gel electrophoresis is shorter than the relaxation time *T*_f_ for the transient free-solution electrophoresis by a factor Ω_s_ and becomes shorter as *λa* decreases. This is because the steady gel electrophoretic mobility *μ*_s_ itself becomes smaller as *λa* increases [36], and the time required to reach the steady value becomes smaller as *λa* increases. The dotted curves (*λa* = 10, and 100) are the results calculated via Equation (72) for the large *λa* approximate gel electrophoretic mobility.

## 4. Conclusions

We have derived an approximate expression (Equation (63)) and its approximate form with negligible errors (Equation (66)) for the Laplace transform $\hat{\mu }\left(s\right)$ of the transient gel electrophoretic mobility *μ*(*t*) of a sphere in a polymer gel medium. Equations (63) and (66) are the generalization of the result of Saad and Faltas [40] and are applicable for low zeta potentials and arbitrary values of *κa*. Equation (66), in particular, which does not involve exponential integrals, is convenient for practical use. It is shown that the relaxation time *T* for the transient gel electrophoretic mobility *μ*(*t*) to reach its steady value *μ*_s_ is shorter than that for the transient free-solution electrophoretic mobility, and *T* becomes shorter as *λa* increases. 

## Data Availability

Not applicable.
